# Characterization of Human DNA Polymerase Delta and Its Subassemblies Reconstituted by Expression in the Multibac System

**DOI:** 10.1371/journal.pone.0039156

**Published:** 2012-06-18

**Authors:** Yajing Zhou, Xiao Meng, Sufang Zhang, Ernest Y. C. Lee, Marietta Y. W. T. Lee

**Affiliations:** 1 Institute of Life Sciences, Jiangsu University, Zhenjiang, Jiangsu, People's Republic of China; 2 Department of Biochemistry and Molecular Biology, New York Medical College, Valhalla, New York, United States of America; University of Hawaii Cancer Center, United States Of America

## Abstract

Mammalian DNA polymerase δ (Pol δ), a four-subunit enzyme, plays a crucial and versatile role in DNA replication and DNA repair processes. We have reconstituted human Pol δ complexes in insect cells infected with a single baculovirus into which one or more subunits were assembled. This system allowed for the efficient expression of the tetrameric Pol δ holoenzyme, the p125/p50 core dimer, the core+p68 trimer and the core+p12 trimer, as well as the p125 catalytic subunit. These were isolated in milligram amounts with reproducible purity and specific activities by a highly standardized protocol. We have systematically compared their activities in order to gain insights into the roles of the p12 and p68 subunits, as well as their responses to PCNA. The relative specific activities (apparent *k*_cat_) of the Pol δ holoenzyme, core+p68, core+p12 and p125/p50 core were 100, 109, 40, and 29. The corresponding apparent *K*_d_'s for PCNA were 7.1, 8.7, 9.3 and 73 nM. Our results support the hypothesis that Pol δ interacts with PCNA through multiple interactions, and that there may be a redundancy in binding interactions that may permit Pol δ to adopt flexible configurations with PCNA. The abilities of the Pol δ complexes to fully extend singly primed M13 DNA were examined. All the subassemblies except the core+p68 were defective in their abilities to completely extend the primer, showing that the p68 subunit has an important function in synthesis of long stretches of DNA in this assay. The core+p68 trimer could be reconstituted by addition of p12.

## Introduction

Three eukaryotic DNA replicative polymerases, Pol α, Pol δ, and Pol ε, are involved in chromosomal DNA replication. RNA-DNA primers synthesized by Pol α/primase are elongated by Pol δ and/or Pol ε [1]. Pol δ requires the DNA sliding clamp, PCNA (Proliferating Cell Nuclear Antigen) [2], for highly processive enzyme activity. In yeast, Pol δ is able to function as a leading strand as well as a lagging strand polymerase in the absence of Pol ε catalytic activity [3]. Synthesis of the SV40 genome in an *in vitro* mammalian replication system can be performed by Pol δ without the presence of Pol ε [4]. However, studies in yeast support a model where there is a division of labor, in which Pol ε synthesizes most of the DNA on the leading strand template while Pol δ synthesizes most of the lagging strand DNA [5], [6]. In addition to its crucial role in DNA replication, Pol δ is also a major participant as a gap-filling polymerase in DNA repair processes and in homologous recombination [1], [7], [8].

Pol δ was initially characterized by its isolation from calf thymus [9], [10] and human placenta [11] as a tightly associated heterodimer of a 125 kDa catalytic subunit which contains both the polymerase and exonuclease catalytic domains [12] and a 50 kDa subunit. This dimer forms the core enzyme, and it was later shown that mammalian Pol δ has two additional subunits, p68 [13], [14] and p12 [15]. The p68 is attached to the core enzyme via and interaction with p50, and p12 forms a bridge between p125 and p50 [16], [17]. In *S. cerevisiae*, Pol δ is a three-subunit enzyme, lacking a counterpart of the p12 subunit [18], while in *S. pombe*, Pol δ is also a four-subunit enzyme [19]. PCNA, the DNA sliding clamp that is required for Pol δ processivity, binds numerous other proteins that participate in DNA transactions involving replication and repair, was originally discovered through its effects on Pol δ activity [20]. All four mammalian Pol δ subunits have been reported to interact with PCNA. Human p125 was the first subunit to be shown to interact with PCNA [21], [22]; subsequently, there were reports for the interaction of p50 [23], p68 [13], [24] and p12 [16] with PCNA. The interaction of p50 with PCNA appears to be much weaker than that of the other three subunits [25]. An assessment of the role of these subunits in PCNA interaction by comparison of the response Pol δ and its subassemblies in a quantitative manner has not been previously reported.

The development of baculovirus systems for the expression of human Pol δ in insect cells has facilitated studies of the Pol δ holoenzyme (Pol δ4) and its subassemblies, *viz*., the p125/p50 core dimer, the core+p68 and core+p12 trimers. These studies have provided some insights into the contributions of the non-catalytic subunits through the characterization of the recombinant Pol δ subassemblies [16], [26], [27]. These previous studies indicated that the core+p12 was as active as Pol δ4 in the standard assay on poly(dA)/oligo(dT) in the presence of PCNA, but was defective in the M13 assay which uses a singly primed ssM13 template and requires RPA (replication protein A), RFC (Replication Factor C) and PCNA [16]. The core+p68 trimer had low activity (7–20% of the holoenzyme) and was defective in the M13 assay [16], [27]. The Pol δ core (p125/p50) also exhibited low activity, was poorly responsive to PCNA, and strikingly had almost no activity in the M13 assay [26]. This was surprising since most of the earlier studies of mammalian Pol δ were of the core dimer which did have activity in the M13 assay, and suggested that the core enzyme could not be properly reconstituted in the insect expression system in the absence of at least the p12 subunit.

Maintaining high-fidelity chromosomal DNA replication is essential for the preservation of genomic integrity and avoidance of the mutations that can lead to disease. Mammalian cells respond to DNA damage by a host of defense mechanisms that include activation of cell cycle checkpoints and DNA repair mechanisms [28], [29]. Among these responses, it has been found that the p12 subunit is degraded when cells are subjected to DNA damage or replication stress, converting Pol δ from the tetrameric form to the trimeric form consisting of the core+p68 [30]. These findings raise a number of questions about the fundamental properties of the core enzyme and what contributions of the p12 and p68 subunits make to PCNA-dependent functions of Pol δ in DNA replication. In this work, we describe the use of an expression system that allows the reconstitution of the core p125/p50 dimer, the core+p68 and the core+p12 in active forms. This has allowed us to compare the enzyme activities of Pol δ and its subassemblies (the core dimer and the two trimers), as well as their binding affinities for PCNA, which has not been examined all together in the same experiments before.

## Materials and Methods

### Materials

All reagents and chemicals used in this study were purchased from Sigma-Aldrich (St. Louis, MO), GE Healthcare (Piscataway, NJ), Gibco-BRL, and Invitrogen except as otherwise indicated. Sf9 cells (SFM adapted) were obtained from Invitrogen (Life Technologies). HeLa cells were obtained from ATCC.

### Generation of Recombinant Baculoviruses by the MultiBac System

The coding regions for the human Pol δ subunits p125, p50, p68, and p12 between the *Bam*HI and *Xba*I sites in the pCDNA3.1(+)-FLAG vector [16] were excised and subcloned into the MCS1 multiple cloning site of the transfer vector pFBDM (kindly provided by Dr. T.J. Richmond, Institute for Molecular Biology and Biophysics, ETH Zürich, Switzerland), in which each subunit was under the control of an individual *polyhedrin* gene promoter. The recombinant transfer vectors with different subunit assemblies were generated according to “MultiBac Expression System User Manual” [31]. The generated recombinant transfer vectors containing multi-subunit gene expression cassettes were introduced into MultiBac baculoviral DNA in DH10MultiBac^Cre^ *E. coli* cells which contain the factors for Tn7 transposition. Recombinant bacmids were generated in cells through the transposition of the Tn7 elements from the pFBDM derivative to the mini-attachment Tn7 target site on the bacmid DNA. Colonies containing bacmid carrying integrated cassettes were identified by blue/white screening and PCR analysis. Bacmid DNAs were prepared from selected white phenotype clones and used for the transfection of insect cells for the generation of viral stocks. Preparation of bacmid DNAs and transfection of Sf9 cells were carried out according to protocols in the Bac-to-Bac^TM^ Baculoviorus Expression Systems Manual (Invitrogen, Life Technologies Incorporated, 2000).

Using the methods described in [31], each single subunit was inserted into the MCS1 multiple cloning site of pFBDM between the *Bam*HI and *Xba*I sites to generate pFBDM-p125, pFBDM-p50, pFBDM-p68 and pFBDM-p12, respectively. The entire expression cassettes were then excised by *Pme*I and *Avr*II digestion and inserted into the multiplication module of a pFBDM derivative containing further gene(s) via *Spe*I/*Bst*Z17I sites. *Spe*I produces a cohesive end compatible with *Avr*II, while *Bst*Z17I and *Pme*I are blunt-cutters. The involved restriction sites were eliminated in the process and multiplication was repeated iteratively using the module present in the inserted cassette. Thus, we constructed recombinant transfer vector pFBDM-[p125/p50/p68/p12] and a set of subassemblies, pFBDM-[p125], pFBDM-[p125/p50], pFBDM-[p125/p50/p68], and pFBDM-[p125/p50/p12]. For viral stock preparation, constructed pFBDM derivatives were introduced into MultiBac baculoviral DNA in DH10MultiBac^Cre^ *E. coli* cells by Tn7 transposition. Isolated Bacmid DNAs from the white clones, identified by blue/white screening and PCR analysis, were transfected into Sf9 insect cells. Recombinant baculoviruses for the catalytic subunit p125, the Pol δ core (p125/p50), the two trimers (core+p68 and core+p12), and the holoenzyme (Pol δ4) were generated by this method.

### Plasmid Construction for His-tagged subunits

The construction vectors for expression of His-tagged individual subunits was as previously described [16]. For wild type His-tagged p125 and p50, the full-length p125 or p50 cDNA sequence was inserted between the *Nde*I and *Bam*HI sites of the pET33b vector, respectively. For wild type His-tagged p12, the full-length p12 cDNA sequence was inserted between the *Nde*I and *Bam*HI sites of the pET15b vector. While for wild type His-tagged p68, the full-length p68 cDNA sequence was inserted between the *Nde*I and *Hin*dIII sites of the pTacTac vector with eight histidine residues added at its N terminus.

### Purification of His- or GST-tagged Proteins

His-tagged proteins of His-p125, His-p50, His-p68, and His-p12, expressed in *E. coli* BL21DE3(plys), were purified by the use of nickel-nitrilotriacetic acid agarose (Qiagen) and further purified by ion exchange chromatography on a FPLC Mono Q column (GE Healthcare) as previously described [16]. GST-tagged p12 in the pGEX-5X-3 vector (GE Healthcare) was expressed in *E. coli* BL21DE3(plys), and purified on glutathione beads (Amersham Biosciences) [16]. Non-tagged p12, used in reconstitution assays, was then released by proteolysis with Factor Xa, and the glutathione S-transferase was removed with glutathione-Sepharose.

### Purification of Recombinant Human PCNA

Human PCNA expressed in *E. coli* was purified using conventional chromatography as described previously [32], with minor modifications. The pTACTAC vector harboring human PCNA was expressed in one liter of DH5-α cell. Harvested cells were disrupted by sonication in lysis buffer (25 mM Tris-HCl, 1 mM EDTA, 25 mM NaCl, 0.01% Nonidet P-40, 2 mM benzamidine, 2 mM pepstatin A, 1 mM PMSF, 1 mM DTT, 100 µg/ml lysozyme, pH 7.4). After centrifugation, the supernatant was chromatographed on a Q-sepharose column. The peak fractions containing PCNA were identified by Western blotting, pooled, dialyzed against TGEED buffer (40 mM Tris-HCl, pH 7.8, 10% glycerol, 0.5 mM EDTA, 0.1 mM EGTA, 1 mM dithiothreitol), and further purified on a 4 ml Mono-P HR 5/20 column.

### Expression and Purification of Recombinant Human Pol δ and its Subassemblies

A 600 ml suspension culture of Sf9 insect cells at 2×10^6^ cells/ml was infected with recombinant baculoviruses at MOI of 2 for 72 hours. The cells were collected by centrifugation at 3,000 rpm for 5 minutes. The cell pellet was suspended in lysis buffer (20 mM Tris-HCl, pH 7.8, 10% glycerol, 0.5 mM EGTA, 1 mM EDTA, 1 mM MgCl_2_, 50 mM NaCl and 0.01% NP-40, protease inhibitor mixture, 1 mM phenylmethylsulfonate) on ice for 30 minutes, followed by sonication using 4×15 second bursts with a 15 second cooling period between each burst and centrifugation at 15,000 rpm for 45 minutes. The supernatant was mixed with 10 ml of 78F5 anti-p125 immunoaffinity agarose beads [33] with end to end rotation overnight and then loaded into a column. The column was washed with 10-bed volumes of TGEE (40 mM Tris-HCl pH 7.8, 10% glycerol, 0.5 mM EDTA, 0.1 mM EGTA) buffer containing 0.1 M NaCl. Pol δ (or its subassemblies) was eluted using 5 bed volumes of TGEE containing 0.4 M NaCl and 30% ethylene glycol. The eluted fractions were assayed for Pol δ activity by the incorporation of [^3^H]dTTP into poly(dA)/oligo(dT) template-primer in the presence of PCNA. The peak fractions were pooled, adjusted to a conductivity corresponding to 100 mM NaCl in TGEED buffer, and loaded onto a 1 ml Mono Q column equilibrated with TGEED buffer. The column was washed with 2 bed volumes of TGEED buffer and eluted with 20-bed volumes of a linear gradient of NaCl from 0.1 to1 M in TGEED buffer. The purity of the fractions was evaluated by 12.5% SDS-PAGE and Coomassie blue or silver staining. The specific activities of recombinant Pol δ holoenzyme preparations prepared by this method were found to be highly consistent, at about 20,000 units/mg protein when assayed on poly(dA)/oligo(dT) in the presence of PCNA under the standard assay conditions (Materials and Methods), and where one unit was defined as 1 nmole dTTP incorporated per hour at 37°C.

### Immunoaffinity Purification of the Native Pol δ Holoenzyme and the Core+p68 Trimer from HeLa Cells

A protocol for the UV treatment of the HeLa cells and subsequent immunoaffinity purification of Pol δ complexes was used essentially as described for their isolation from HEK 293T cells [30]. HeLa cells were grown to about 80% confluence in 100 plates (150×25-mm). Half of the plates were treated with UV-C (20 J/m^2^) and harvested 4 hours later while the other half was harvested with no treatment. All following purification steps were carried out at 4°C. The pelleted cells were lysed, disrupted by sonication, and equal amounts of total protein in the centrifuged supernatants from the UV-C treated and the untreated cells were chromatographed on individual columns. Each column contained 5 ml of anti-p125-agarose beads. The two columns were run in parallel under the same conditions. The columns were washed with 10 bed volumes of TGEE buffer containing 0.1 M NaCl and then eluted using 5 bed volumes of TGEE containing 0.4 M NaCl and 30% ethylene glycol. Fractions of 0.3 ml each were collected. Assays of the column fractions for Pol δ activity and other analyses were performed in parallel with minimum delay.

### Reconstitution of Pol δ from the Core+p68 Trimer with Recombinant p12

Recombinant core+p68 was pre-incubated with different concentrations of non-tagged p12 at 4°C for 30 min before assay for the restoration of Pol δ activity either on poly(dA)/oligo(dT) primer-template or singly primed M13 DNA template as described below. The native trimer lacking p12 used as a comparison was isolated from UV- treated HeLa cells.

### DNA Polymerase Assays

The standard assay for Pol δ activity was performed using poly(dA)/oligo(dT) as previously described [16], [30]. The reaction mixture contained 0.25 OD unit/ml of sparsely primed poly(dA)_4000_/oligo(dT)_50_ (Supertechs, Bethesda, MD) in 50 mM Hepes pH 6.5, 5% glycerol, 0.1 mg/ml BSA, 5 mM MgCl_2_, 5 μM dTTP and 0.5 μM [^3^H]dTTP. The reactions were started by adding 100 ng of recombinant human PCNA and ∼0.2 units of Pol δ in a total volume of 30 µl, followed by incubation at 37°C for 30 minutes. The reactions were terminated by spotting onto DE81 papers which were washed 3 times with 0.3 M ammonium formate, pH 7.8 and once with 95% ethanol, dried and counted using a liquid scintillation counter. One unit of DNA polymerase activity corresponds to the incorporation of 1 nmole of dTMP per hour at 37°C. Assays using singly primed M13 DNA as the template were performed as previously described [16], [30]. Single stranded M13mp18 DNA (7250 bp, New England Biolabs) was primed with a 20-mer oligonucleotide (5′-CTAGAGGATCCCCGGGTACC-3′) complementary to nucleotides 6262-6243 of the M13 genome. Standard reactions contained 40 mM Tris-HCl, pH 7.8, 1 mM DTT, 0.2 mg/ml BSA, 10 mM MgCl_2_, 0.5 mM ATP, 50 mM NaCl, 250 µM each of dTTP, dCTP, and dGTP, 25 µM dATP, 3 µCi of [α-^32^P] dATP, 100 ng of primed M13 template, 80 ng RFC, 200 ng of RPA, and variable amounts of Pol δ and 100 ng of recombinant human PCNA in a 30 µl reaction volume. The reaction mixtures were incubated at 37°C for 30 minutes and were terminated by the addition of 20 mM EDTA. Aliquots (5 μl) of each reaction were spotted onto DE81 papers which were washed 3 times with 0.3 M ammonium formate pH 7.8, once with 95% ethanol, dried and counted using a liquid scintillation counter. The remainder of the products were run on a 1.5% alkaline agarose gel at 50 V for 2.5 hours. The gel was dried and the products were visualized with a phosphoimager or evaluated on an x-ray film. Where two Pol δ enzymes were compared the samples were run on the same gel, or visualized under identical conditions where they were run on separate gels. For quantitation of incorporation samples were spotting onto DE81 papers, washed and counted as described above.

### Western Blot Analysis

A western blotting protocol, monoclonal antibodies against p125 (78F5), p50 (13D5 and 17D2), and rabbit polyclonal antibodies against p68 and p12 were as described previously [17]. The Pol δ subunits were separated by 12.5% SDS-PAGE gels and transferred to a nitrocellulose membrane. The membrane was then stained with Ponceau S and cut into four pieces according to the molecular weight of each subunit. The four pieces of membrane were blocked with 5% w/v nonfat dry milk in TBST buffer (20 mM Tris-HCl, pH 7.8, 150 mM NaCl, 0.05% Tween 20) for 1 hour at room temperature. The blots were then incubated with individual primary antibody corresponding to each subunit for 1 hour at room temperature or overnight at 4°C. After three 15 min washes in TBST, the blots were incubated with HRP-conjugated goat anti-mouse IgG or goat anti-rabbit IgG (Pierce) for 1 hour and washed with TBST 3 times for 10 min. Super Signal West Pico chemiluminescence substrate (Pierce) was used for signal production.

### Cleavage of Recombinant Pol δ by Human Calpain-1 in vitro

The cleavage reaction mixture in a total volume of 20 μl contained 480 ng purified recombinant Pol δ4, 40 mM Tris-HCl, pH 7.5, 120 mM NaCl, 6 mM CaCl_2,_ and 1 unit of human calpain-1 (BioVision, Inc). Calpain-1 inactivated by incubation at 100°C for 10 min was used as a control. Incubations were at 30°C for 1 hour. N-Acetyl-Leu-Leu-Nle-CHO (ALLN) or calpeptin, where added, were 26 μM or 5 μM, respectively. The generated products were separated by 12.5% SDS-PAGE gel and transferred to nitrocellulose membranes for western blot analysis.

### Protein Determination

Protein concentrations were determined by the Bradford method with bovine serum albumin as a standard, or by “in-gel” determination of the catalytic subunit p125 concentration using catalase as a protein standard.

## Results

### Expression of Human Pol δ Complexes with the MultiBac Vector System and Their Isolation from Infected Insect Cells

Our understanding of the fundamental biochemical properties of mammalian/human Pol δ lags behind those of replicative polymerases in other systems. Much of the information regarding the biological functions of Pol δ has come from studies in yeast systems, particularly in *S. cerevisiae*. A major obstacle is the availability of highly purified active mammalian Pol δ. Extensive studies of calf thymus and human Pol δ led to its characterization as a heterodimer, a core enzyme of the p125 and p50 subunits [9], [11]. Development of baculovirus expression systems using vectors for the individual subunits facilitated the studies of human Pol δ since this allowed reconstitution of the recombinant Pol δ enzyme and its subassemblies in insect cells [17], [27]. In our hands, there were problems of consistency in the quality and yields of the preparations, and some discrepancies in the expected properties of the subassemblies, particularly of the core dimer. One potential source of the problem was that these methods depend on the co-infection of the insect cells with multiple recombinant baculoviruses each containing an individual subunit. To avoid the necessity for co-infection with mixtures of recombinant baculoviruses, we took advantage of the MultiBac expression system which is designed for production of multiprotein eukaryotic complexes [31], [34]–[36]. This allowed for the facile construction of single baculovirus vectors into which all four Pol δ subunit cDNAs were inserted. Thus, recombinant baculoviruses for the catalytic subunit p125, the Pol δ core (p125/p50), the two trimers (core+p68 and core+p12), and the holoenzyme (Pol δ4) were generated (Materials and Methods).

We used a highly standardized protocol for rapid isolation of recombinant Pol δ heterotetramer and its subassemblies through immunoaffinity chromatography and FPLC Mono Q chromatography. For maximal yields and stability the purifications were performed within 48 hr, and the preparations were stored at high protein concentration in liquid nitrogen. This procedure allowed the purification of the Pol δ complexes to near-homogeneity. Routinely, as much as 3–4 mg of protein complexes could be obtained from 300 ml of infected Sf9 cells.

One of the difficulties in isolation of Pol δ from mammalian tissues is the loss of the p68 subunit which is prone to proteolytic nicking [13], [15]. The MultiBac system [31], [34]–[36] uses an engineered baculovirus genome in which two baculovirus genes, *v-cath* which encodes for a viral protease V-CATH which is activated upon cell death by a process dependent on a juxtaposed gene on the viral DNA, and *chiA* which encodes for a chitinase, were disrupted. Therefore, in our work, the quality of proteins produced with MultiBac system was significantly improved through a reduction of viral-dependent proteolytic activity and reduced cell lysis. No degradation of the p68 subunit was observed in our preparations as judged by Coomassie Blue or silver stained SDS-PAGE gels (data not shown).

**Figure 1:**
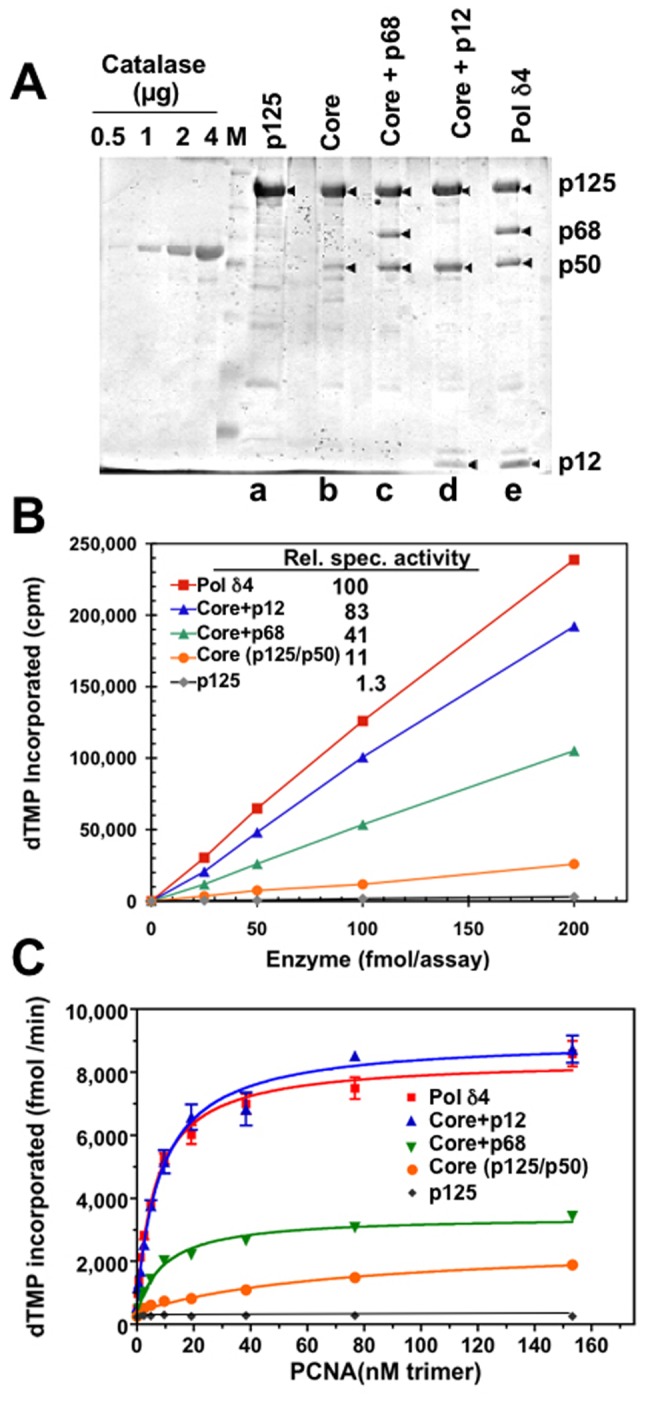
Comparison of the activities and PCNA stimulation of Pol δ and its subassemblies on poly(dA)/oligo(dT) primer-templates. Panel A. “In-gel” determination of p125 content of each Pol δ assemblies. The recombinant p125 alone (lane a), core enzyme of p125/p50 (lane b), core+p68 trimer (lane c), core+p12 trimer (lane d), and Pol δ4 (lane e) were expressed and purified to near-homogeneity by immunoaffinity chromatography and FPLC on Mono-Q ion exchange as described in “Materials and Methods”. Protein concentrations were determined based on the in-gel analysis of p125 content for each of the complexes using catalase as a protein standard. The peak fraction of each enzyme from the Mono Q step was separated on 12.5% SDS-PAGE and followed by silver staining. The four left lanes show the catalase standards (0.5, 1, 2, and 4 μg). Lane “M” contains the protein markers in kDa. The four subunits are marked by arrows. Panel B. Pol δ activity assay. Increasing amounts of each enzyme in fmoles of p125 per reaction were analyzed for their activities in the standard poly(dA)/oligo(dT) assay in the presence of PCNA. The vertical axis indicates the incorporated dTMP in CPMs and the horizontal axis shows the enzyme concentrations in fmoles of p125. Panel C. PCNA stimulation. The graph shows the activities (in triplicate) on poly(dA)/oligo(dT) with increasing PCNA. The vertical axis indicates the incorporated dTMP in fmoles per minute and the horizontal axis shows the PCNA concentrations in nM trimer. The recombinant enzyme complexes are indicated by the lines.

We used preparations of p125, Pol δ core, core+p68, core+p12, and the Pol δ4 holoenzyme for comparison of their functional properties. Protein stained SDS-PAGE gels of typical preparations are shown in Figure 1A. To insure that changes in activity due to stability did not influence our data, the preparations were assayed at the time of preparation as well as before use in the standard assay using poly (dA)/oligo(dT). In addition, we used of an “in gel” system for determination of the protein content of p125 with catalase as an internal protein standard (Fig. 1A). This was done so that all the preparations could be compared in terms of their p125 protein content, rather than total protein, thereby minimizing errors due to the presence of small amounts of protein impurities.

### Comparison of the Specific Activities of the Pol δ Enzymes on Poly (dA)/oligo(dT) Template/primer

The activities of Pol δ enzymes were compared using poly (dA)/oligo(dT) as the template/primer in the commonly used assay for Pol δ activity. The template/primer used was a poly (dA)_4000_ homopolymer sparsely primed with an oligo(dT)_50_ primer (ca. 9:1). In this assay system Pol δ activity is dependent on PCNA which promotes processive DNA synthesis [17], [20]. The assays of product formation with increasing protein concentration (based on content of p125) are shown in Figure 1B. From the slopes of the linear curves the specific activities (moles product/moles p125 content per unit time) were determined. Relative to the Pol δ4 holoenzyme taken as 100, these were as follows: p125 catalytic subunit 1.3; core enzyme (p125/p50), 11; core+p68, 41; core+p12, 83. The specific activities of the holoenzyme and the core+p12 trimer were similar to those of reconstituted enzymes that we and others have previously reported using individual baculovirus vectors for their expression in insect cells [17], [27], [37]. The core+p68 trimer exhibited ca. 40% of the activity of the holoenzyme, unlike earlier reports which indicated that it has very low activity [17], [27], [37] (The basis for this discrepancy is likely due to its stability on storage, as shown later in this report.). The recombinant core dimer, which previously had been found to have little or no activity, had ca. 10% of the activity of the holoenzyme. Thus, we have for the first time reconstituted a recombinant core enzyme that is active like the native Pol δ dimer isolated from calf thymus and human tissues [9], [11]. As previously observed, the p125 subunit exhibits very little activity (*ca*. 1% relative to Pol δ4) [16], [26], [27].

### Comparison of the PCNA Stimulation of Pol δ and its Subassemblies

The responses of Pol δ4 and its subassemblies to increasing concentrations of PCNA were determined. The data for product formation with increasing PCNA concentrations are shown in Figure 1C. This allowed the determination of the PNCA concentrations required for half-maximal stimulation of Pol δ activity. These were taken as the apparent *K*_d_'s for PCNA, and the maximal activities were taken as the *k*_cat_ values in terms of specific activities (moles dTMP incorporated/mole p125/min) (Fig. 1C). The comparisons of the apparent *K*_d_ for PCNA, apparent *k*_cat_, and relative *k*_cat_ values are summarized in Table 1.

**Table 1:** Comparison of the Apparent *k*_cat_ and *K*_D_ Values For PCNA of Pol δ Assemblies^a^.

	Polymerase Activity		
Pol δ assemblies	*k*_cat_ (min^−1^)	Relative *k*_cat_	PCNA (*K*_d_, nM trimer)
Pol δ4	77±2.5	100	7.1±1.0
core+p12	83±2.6	109	8.7±1.2
core+p68	31±1.5	40	9.3±1.9
core	22±2.7	29	73±23
p125	⋅⋅⋅	⋅⋅⋅	⋅⋅⋅

a Data from the assays of Pol δ complexes on poly(dA)/oligo(dT) template/primers shown in Figure 1C were fitted into the hyperbola binding equation, *v* = *v*_basal_+{V_max_•[PCNA]/(*K*_d_+[PCNA])} where *v* is the velocity for dNMP incorporation; *v*_basal_ is the dNMP incorporation without PCNA; *K*_d_ is the apparent dissociation constant of PCNA. The apparent *k*_cat_ was calculated as *V*_max_/E. The relative specific activities (relative *k*_cat_) were determined by taking Pol δ4 as 100.

Analysis of the *K*_d_'s for PCNA shows that Pol δ4 has a *K*_d_ of *ca*. 7 nM PCNA trimer, but surprisingly, both the core+p68 and core+p12 trimers had *K*_d_'s of *ca.* 9 nM, *i.e*., the loss of p12 or p68, both of which have PCNA binding abilities, did not alter the apparent affinities for PCNA, contrary to expectation. In contrast, the core enzyme had a 10 fold higher *K*_d_ for PCNA, of *ca*. 70 nM. Addition of either p68 or p12 is sufficient to maximize the binding of the Pol δ core to PCNA, based on their activities on the poly (dA)/oligo(dT) template/primer.

The relative *k*_cat_ values for Pol δ4, core+p12 and core+p68 are similar to the data based on specific activities in the routine Pol δ assay (Fig. 1B) as PCNA was added at saturating levels (40 nM). The core+p12 enzyme has a relative *k*_cat_ equivalent to that of the holoenzyme, while that of the core+68 is 40% of that of the holoenzyme. The core enzyme has relative *k*_cat_ of 29% that of the holoenzyme. This is higher that the relative specific activity of 11% determined in the standard assay, and is explained by the fact that its affinity for PCNA is *ca*. 10 fold weaker than that of Pol δ4.

Thus, our data support a perspective in which the core enzyme has sufficient affinity for PCNA to carry out elongation of the oligo (dT) primer, and that addition of either the p68 or p12 subunit is sufficient to increase the affinity for PCNA so that a maximal stimulation is observed. However, interpretation of these data must bear in mind that these are derived from measurement of the response of the enzymes to PCNA with the poly (dA)/oligo(dT) template primer. While Pol δ performs processive synthesis in the presence of PCNA in this assay, comparatively short stretches are synthesized, and the template is in excess of the enzymes.

### Comparison of Processive DNA Synthesis by the Pol δ Enzymes on Singly Primed M13 DNA

Next we compared the activities of the Pol δ enzymes in the singly primed M13 DNA assay. In this assay a singly primed 7.4 kb single stranded circular M13 DNA is used, and the Pol δ concentration used is in slight excess of the template/primer. The assay requires the loading of PCNA with RFC, and the addition of RPA, a single stranded DNA binding protein, to coat the ssDNA. This assay has been extensively used to examine the abilities of Pol δ to perform processive DNA synthesis on large templates and provides a more demanding test for defects in Pol δ function than the poly (dA)/oligo(dT) assay. Pol δ is able to complete the synthesis of the 7.4 kb M13; however, this may involve dissociation of the enzyme from the template, *i.e.*, the M13 DNA is not synthesized in a single pass [27].

**Figure 2:**
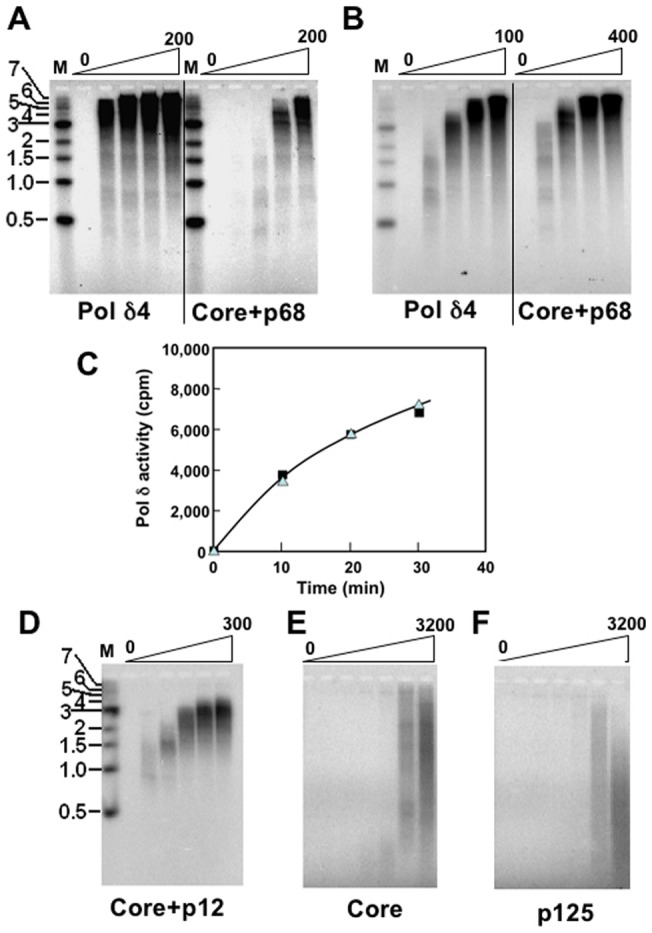
Analysis of DNA products synthesized by Pol δ and its subassemblies on primed M13 DNA. The assays contained 3 µCi each of [α-^32^P]dATP, 100 ng of primed M13 template with RFC, RPA, and variable amounts of enzymes and 100 ng of PCNA in a total 30 µl reaction volume as described in “Materials and Methods”. After incubation for 30 min at 37°C, the reaction products were loaded onto a 1.5% alkaline agarose gel, electrophoresed, dried, and visualized by phosphorimaging. Horizontal bars on the left indicate the positions in kb of the DNA markers (“M”). Panels A, B. The activities of core+p68 and Pol δ4 were determined as described above. In panel A the lanes from left to right show product formation with increasing amounts of enzyme (0, 25, 50, 100, 200 fmoles). In panel B the experiment was performed as in panel A except that the enzyme amounts were adjusted to 0, 12.5, 25, 50, 100 fmoles for Pol δ4 and 0, 50, 100, 200, 400 fmoles for core+p68. Panel C. The activities of core+p68 and Pol δ4 were determined on primed M13 DNA with CPMs. 100 fmoles of Pol δ4 (solid squares) or 400 fmoles of core+p68 (solid triangles) were assayed on primed M13 DNA and counted with CPMs by a liquid scintillation counter at indicated reaction time (horizontal axis). The vertical axis indicates CPMs incorporated.Panel D. The activities of core+p12 on primed M13 DNA were determined. The lanes from left to right show the products with increasing amounts of enzyme (0, 25, 50, 100, 200, 300 fmoles). Panels E, F. The activities of the core enzyme and p125 subunit respectively were determined. The lanes from left to right show product formation with increasing amounts of enzyme (0, 25, 50, 100, 400, 800, 3200 fmoles for both enzymes).

The products formed by the Pol δ enzymes were analyzed by alkaline agarose gel electrophoresis (Fig. 2). In this system, both the Pol δ trimers and the core enzyme exhibited differing degrees of defective function by comparison to the holoenzyme. Comparison of the activities of the core+p68 and Pol δ4 with increasing enzyme concentrations shows that the amount of product and the sizes of the product formed by the core+p68 trimer were markedly reduced (Fig. 2A). This could be compensated for by increasing the concentration of core+p68 by four-fold over that of Pol δ4; the amount and size of products formed were then similar (Fig. 2B), which was further confirmed by the counting of CPMs on a liquid scintillation counter at different reaction time (Fig. 2C). This is in contrast to our previous report that both recombinant core+p68 and the native core+p68 isolated from UV treated HEK293T cells were defective in this assay [30]. Since the relative *k*_cat_ of the core+p68 is ca. 40% of Pol δ4 in the poly (dA)/oligo(dT) assay, this suggests that the ability of the core+p68 for processive synthesis is not much different on the M13 template. Our interpretation of the data is that the reduction in apparent processivity based on the size of the products seen in Figure 2A by core+p68 enzyme could equally be due to reduced enzyme activity or an increased dissociation of the enzyme from PCNA.

In the case of the core+p12 trimer, the defects in amount and size of product formed is severe (Fig. 2D) by comparison to Pol δ4 or the core+p68 trimer (cf. Fig. 2A), and could not be compensated for by increasing the enzyme concentration (data not shown). This is in strong contrast to the activity of the core+p12 trimer on poly (dA)/oligo(dT), where it exhibited a robust activity on a par with Pol δ4. Taken together the behavior of the core+p68 enzyme, our findings suggest that p68, more than p12, has a major role in enabling synthesis on the M13 template. Several reasons can be advanced for the defects in the core+p12 reaction that are observed in the M13 assay by comparison to the poly (dA)/oligo(dT) assay. These include a more rapid dissociation of the Pol δ complex from PCNA that comes into play on the longer template. The findings are in contrast to the apparent *K*_d_ values for PCNA obtained from the poly (dA)/oligo(dT) assays (Table 1). However, a more rapid dissociation of the Pol δ subassemblies from PCNA could be masked in the poly(dA)/oligo(dT) assay where PCNA loading is not required and both PCNA and the template are in excess of the enzyme (Fig. 1C). The core enzyme has very poor activity on the M13 template (Fig. 2E) even at a much higher enzyme concentration. The p125 subunit on its own has almost negligible activity on the M13 template (Fig. 2F).

**Figure 3:**
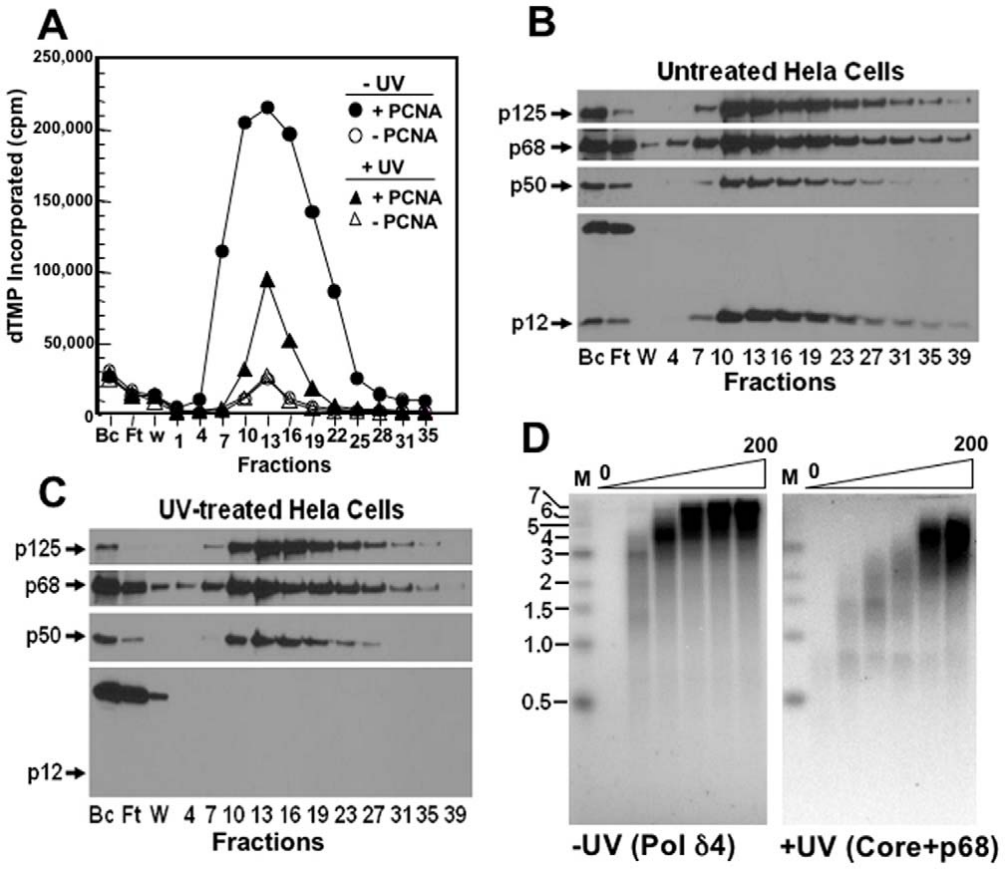
Isolation of Pol δ from UV-treated HeLa cells by immunoaffinity chromatography. HeLa cells (2×10^8^ cells) were grown and divided into two portions, one of which was treated with UV-C (20 J/m^2^ at 254 nm) and harvested 4 hour later and the other was used as the control. The UV-treated and control cells were lysed and purified concurrently on two 5-ml columns of anti-p125-agarose. Panel A. Immunoaffinity chromatography: Elution of Pol δ activity. The column fractions were assayed for polymerase activity using poly(dA)/oligo(dT) as the template-primer in the presence (solid circles), and absence (open circles) of PCNA for the untreated cells and for the UV-treated cells (solid triangles, with PCNA; open triangles, without PCNA). The vertical axis indicates the incorporated dTMP in CPMs and the horizontal axis shows the lysate, flow-through, wash, and column fraction numbers. Panel B. The lysate (Bc), flow-through (Ft), wash (W), and column fractions (0.3 ml each) of the immunoaffinity chromatography of the untreated HeLa cells were Western blotted for p12, p125, p50, and p68 (arrows). The lanes from left to right show the column fraction numbers. Panel C. The corresponding fractions from the immunoaffinity chromatography of Pol δ from the UV treated cells were Western blotted for the Pol δ subunits as for Panel B. Panel D. The abilities of the immunoaffinity purified native Pol δ4 (untreated cells) and core+p68 (UV-treated cells) enzymes were tested for their abilities to elongate singly primed M13 DNA (Materials and Methods); images show the gel electrophoresis patterns for product formation. Horizontal bars on the left indicate the positions in kb of DNA markers (“M”). The lanes from left to right show increasing amounts of enzyme (0, 12.5, 25, 50, 100, 200 fmoles).

Because the core+p68 trimer did not display any major defect in extending the M13 template, we re-examined its behavior using the native enzymes isolated from HeLa cells. Pol δ4 and its core+68 trimer were isolated from HeLa cells by immunoaffinity chromatography (Materials and Methods) as we had previously reported for their isolation from HEK293T cells [30]. The native core+p68 trimer was prepared from HeLa cells in which p12 was depleted by treatment with UV as previously described [30]. The elution of Pol δ from the UV-treated and control untreated cells from the immunoaffinity chromatography columns was monitored by its activity in the absence and presence of PCNA (Fig. 3A), and by immunoblotting for each of the Pol δ subunits (Fig. 3B, 3C). Western blots confirmed that the p12 subunit was absent in the UV-treated cells (Fig. 3C). From the eluted activities, an estimate of the recoveries of activity showed that the UV-treated cells yielded 44% of the amount recovered from the control cells, similar to our previous observations with the core+p68 isolated from HEK293T cells. This is consistent with our observations of the relative *k*_cat_ values for recombinant core+p68 and Pol δ4 (Table 1). We then compared the activities of the HeLa enzymes in the M13 assay (Fig. 3D) by analysis of the product formation by gel electrophoresis. As can be seen, both enzymes were active in processive synthesis, and the data are highly similar to that obtained with the recombinant core+p68 and Pol δ4 (Fig. 2B). From these data we conclude that the recombinant core+p68 trimer closely represents the properties of the native core+p68.

### The Recombinant Core+p68 Trimer Isolated from Sf9 Cells and the Native Core+p68 Trimer Isolated from UV-treated HeLa Cells Are Highly Unstable

**Figure 4:**
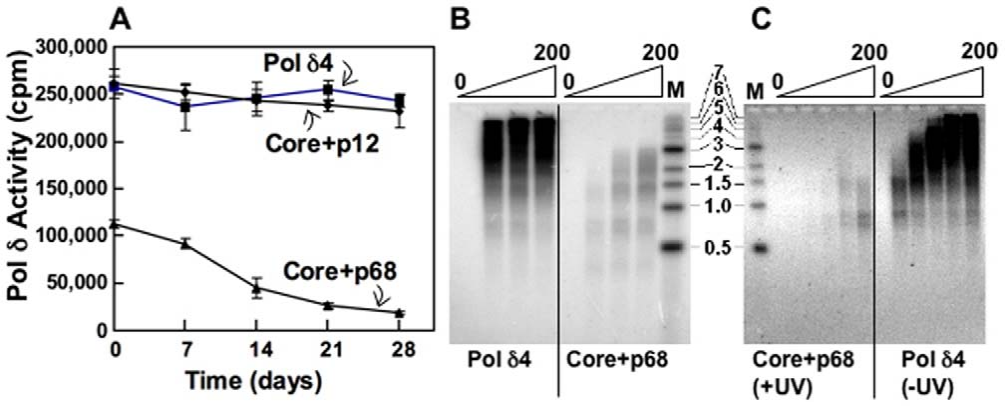
Analysis of the stability of Pol δ holoenzyme and its subassemblies. Highly purified Pol δ enzymes expressed in Sf9 cells or isolated from UV treated HeLa cells as described in “Materials and Methods” were used for the stability examinations. Panel A. Stability of Pol δ enzyme activities assayed using poly (dA)/oligo(dT). Each enzyme (200 fmoles per reaction) was assayed after storage for different time in days. The activities are shown as incorporated dTMP in CPM. Panel B. Activity of recombinant core+p68 on singly primed M13 DNA after four weeks of storage at −80°C. The DNA products synthesized by the recombinant core+p68 were examined on primed M13 DNA with Pol δ4 as a comparison. The lanes from left to right show increasing amounts of enzyme (0, 50, 100, 200 fmoles for both enzymes). “M” indicates the positions in kb of the DNA markers. Panel C. Activity of native core+p68 on singly primed M13 DNA after four weeks of storage at −80°C. The experiment was performed as in panel B except that the core+p68 used was isolated from UV treated HeLa cells (“+UV”) and the Pol δ4 was isolated from untreated cells (“-UV”). The enzyme amounts from left to right were 0, 12.5, 25, 50, 100, 200 fmoles.

The activities of recombinant core+p68 trimer have previously reported to range from 5–20% of that of Pol δ4 [17], [27], [37]. These are lower activities than we observed here, and could be partially explained by the possibility that the core+p68 trimer is highly unstable. The stabilities of newly prepared enzymes, kept at −80°C after the Mono Q step, were examined by the standard assays on poly (dA)/oligo(dT) template/primer and on primed M13 DNA every week over a period of four weeks. The activity of core+p68 lost ∼83% of its activity on poly(dA)/oligo(dT) after four weeks, in contrast to Pol δ4 and core+p12 which were stable under exactly the same conditions (Fig. 4A). Comparison of recombinant Pol δ4 and core+p68 (Fig. 4B) or their native forms isolated from UV-treated HeLa cells (Fig. 4C) on primed M13 DNA after four weeks of storage showed that both recombinant and native core+p68 had lost most of their activity. We found that the instability of the enzymes could be alleviated by a rapid isolation of the enzymes within 48 hr and storage at higher protein concentrations of ca. 1−2 mg/ml in liquid nitrogen.

### Restoration of Pol δ Activity by Reconstitution from the Core+p68 Trimer with Recombinant p12 In Vitro

**Figure 5:**
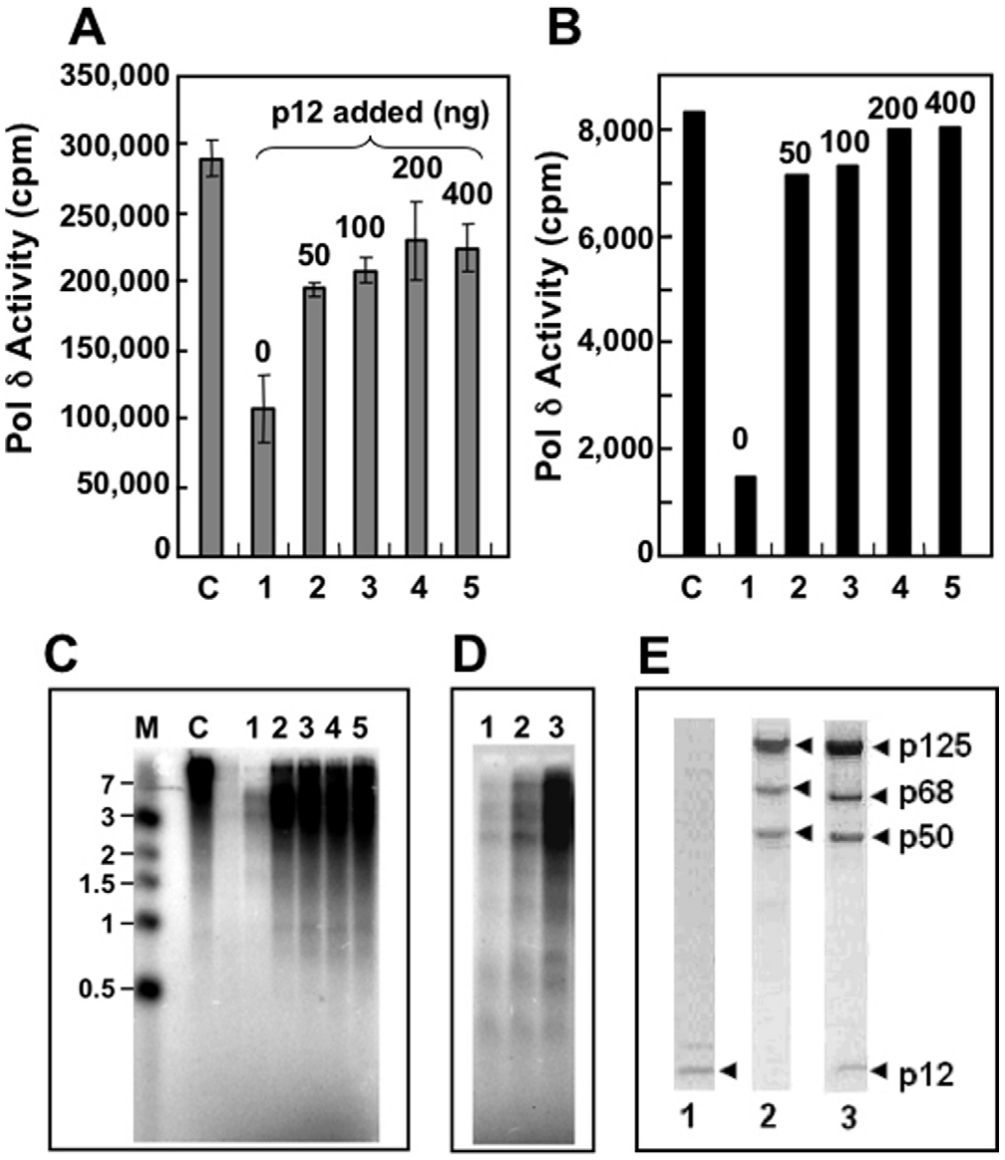
Reconstitution of Pol δ activity by addition of p12 to the core+p68 trimer. Panel A. Recombinant core+p68 (200 fmoles) was pre-incubated with increasing amounts of recombinant p12 at 4°C for 30 min and assayed on poly(dA)/oligo(dT) template-primer. Lane C, 200 fmoles of Pol δ4 alone as a positive control. Lanes 1–5, core+p68 (200 fmoles) preincubated with increasing amounts of p12 (0, 50, 100, 200, 400 ng). Assays were performed in triplicate and the results are shown as cpm incorporated. Panel B. The pre-incubation and enzymes were the same as for panel A except that singly primed M13 was used as the substrate. The amounts of core+p68 or Pol δ4 used were 50 fmoles each, and 50 fmoles of Pol δ4 alone was taken as a control (lane C). Aliquots (5 μl) of each reaction were spotted onto DE81 papers and counted after washing (Materials and Methods). Panel C. Products of the reactions shown in panel B were analyzed on 1.5% alkaline agarose gels. Lane M, DNA markers. Lane C, Pol δ4 control; lanes 1–5, increasing amounts of p12 as for panel B. Panel D. Native core+p68 isolated from UV treated HeLa cells is also stimulated by addition of p12. Reactions were performed as in panel B and the products were examined on a 1.5% alkaline agarose gel. Lanes 1–3, 50 fmoles of native core+p68 was preincubated with 0, 50, 100 ng of p12 as in panel C and the products analyzed on a 1.5% agarose gel. Panel E. Protein stained SDS-PAGE gels of the proteins used. Lane 1, non-tagged p12; lane 2, recombinant core+p68 and lane 3, recombinant Pol δ4.

We and others have previously demonstrated that purified His-tagged p12 could stimulate the activity of recombinant core+p68 or native core+p68 isolated from UV-treated HEK293T cells in poly(dA)/oligo(dT) assay [27], [30]. However, untagged p12 could stimulate the activity of native core+p68 both on poly(dA)/oligo(dT) primer-template and primed M13 DNA [30]. The synthesized products in the M13 assay were in the 3 kb range which suggested an incomplete restoration of processivity of the enzyme. Here, we re-examined the reconstitution of Pol δ activity by addition of recombinant untagged p12 to the core+p68 enzyme. Purified recombinant core+p68 was incubated with p12 and assayed for Pol δ activity on poly(dA)/oligo(dT) in the presence of PCNA. The addition of p12 stimulates the activity of core+p68 and led to ∼80% restoration of activity at saturating amounts of p12 (Fig. 5A). The activity on M13 DNA was also significantly restored to 96% of that of Pol δ4 (Fig. 5B). Analysis of the synthesized products on 1.5% alkaline agarose gels showed p12 restored the processivity of both recombinant and native core+p68 for elongating primers on M13 DNA (Fig. 5C and 5D). The proteins stains for the p12, core+p68 and Pol δ4 preparations used in these experiments are shown in Figure 5E.

**Figure 6:**
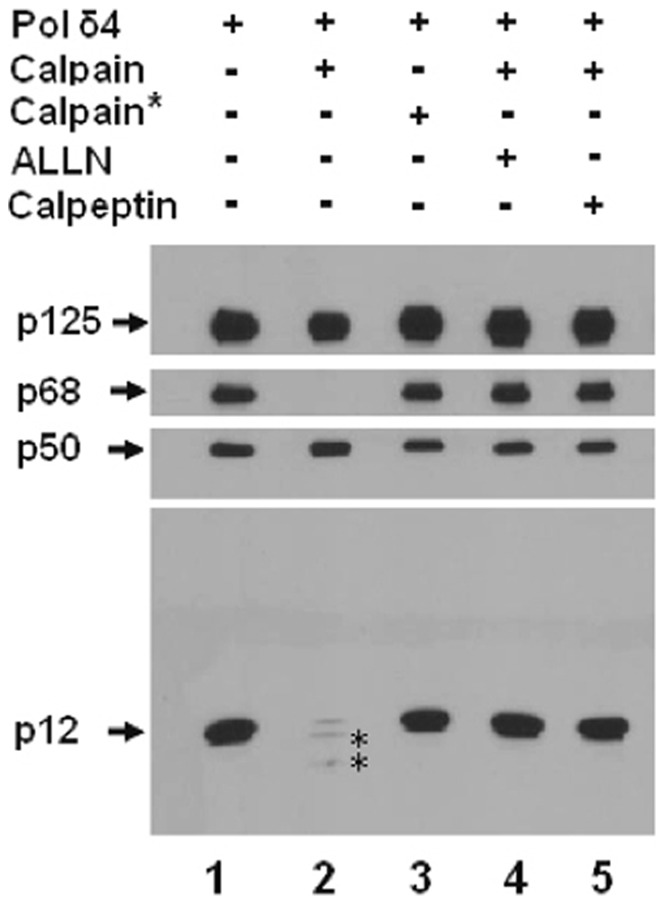
Calpain-1 selectively cleaves the p68 and p12 subunits to convert Pol δ4 to the dimeric core enzyme. Pol δ (480 ng) was incubated with 1 unit of calpain-1 for 1 hr at 30°C as described in “Materials and Methods”. The reactions were analyzed by Western blotting. The four Pol δ subunits are marked by arrows on the left. Lane 1, Pol δ4 negative control. Lane 2, Pol δ4 incubated with human calpain-1 and CaCl_2_. Lane 3, Pol δ4 incubated with heat-inactivated calpain-1 (calpain-1*). Lanes 4 and 5, Pol δ4 incubated with calpain-1 in the presence of the calpain inhibitors ALLN or calpeptin. The truncated fragments of p12 are marked by asterisks.

## Discussion

We have used an improved method for expression of human Pol δ and its subassemblies in Sf9 insect cells by using a single recombinant virus containing one or more of the Pol δ subunits in the MultiBac system. This method allowed the isolation of highly purified enzymes in mg amounts with reproducible purity and specific activities which has allowed us to perform side by side comparisons of the activities and PCNA response of Pol δ complexes in the commonly used standard assays on poly(dA)/oligo(dT) template/primer. Our findings provide some new insights into the properties of the Pol δ complexes. Comparison of Pol δ complexes on poly(dA)/oligo(dT) template-primer indicate that the recombinant core enzyme has significant activity and is PCNA responsive, contrary to previous reports which conflicted with reports of the core enzyme isolated from tissue sources. The first issue was that previous methods yielded a core enzyme that was poorly active or inactive [17], [27], [37], in contrast to the core enzyme isolated from mammalian tissues [9], [11] and the second was that the core+p68 was also poorly active [17], [27]. The likely reason for this discrepancy is that the core+p68 is highly unstable as we demonstrate in this study.

We determined the response of Pol δ and its subassemblies to PCNA and have determined the apparent *K*_d_'s for PCNA using the PCNA concentration dependence of Pol δ activity in the poly(dA)/oligo(dT) assay. We observed that both the trimeric forms had almost the same *K*_d_'s for PCNA as the holoenzyme form, and a drop in *K*_d_ was only observed for the dimeric core enzyme. This was surprising, given that both the p12 and p68 subunits have the ability to bind to PCNA. These findings reflect the complexity of the nature of the interactions of Pol δ with PCNA, since all four of its subunits have the capacity for individually interacting with PCNA. It is likely that Pol δ has multiple modes of interaction with PCNA, which could involve variable combinations of its subunits in multivalent interactions with PCNA. It is also noted that while the individual subunits of human Pol δ are able to interact with PCNA, direct evidence for their involvement by examination of reconstituted holoenzymes in which the PCNA binding motifs have been mutated or inactivated have only been performed for p12 [16], [17] and p68 [38], but not for p125 or p50. However, our findings that the core enzyme has significant activity albeit with a 20 fold higher *K*_d_ for PCNA also establishes that p125 and/or p50 are able to interact with PCNA. In *S. cerevisiae* Pol δ, all three subunits are involved in PCNA binding, and mutations in the PCNA binding motifs have shown that all three subunits are required for optimal PCNA binding [39]. The equivalence of the apparent binding of the two human trimeric subassemblies would suggest that there may be multiple modes of interaction when the Pol δ holoenzyme interacts with PCNA. The requirement for multiple conformations of Pol δ in its binding to PCNA may arise because PCNA serves as a platform on which multiple proteins are involved in coordinated DNA transactions, for example in Okazaki fragment processing, where coordination of Pol δ with Fen1 and other nucleases are required for removal of primers, as well as with DNA ligase for completion of the process [40], [41]. In other systems, structural analysis of PCNA binding proteins that need to coordinate their occupancy of PCNA has provided evidence that these proteins may have multiple conformations of binding with PCNA, *e. g*., in the case of *S. solfataricus* DNA polymerase, Fen1 and DNA ligase interactions with PCNA [42], [43]. However, the future elucidation of the structure of Pol δ will be required to gain further insights into how the individual subunits may physically interact with the PCNA molecule.

Thus, our data using the poly(dA)/oligo(dT) assays support a perspective in which the core enzyme has sufficient affinity for PCNA to carry out elongation of the oligo(dT) primer, and that addition of either the p68 or p12 subunit is sufficient to increase the affinity for PCNA so that a maximal stimulation is observed. However, interpretation of these data must bear in mind that these are derived from measurement of the response of the enzymes to PCNA with the poly(dA)/oligo(dT) template primer. This substrate consists of a sparsely primed 4000 nt poly(dA) template onto which PCNA is freely able slide onto the ends and does not require loading by RFC; the homopolymeric nature of the template also eliminates the possibility of formation of secondary structures that could lead to stalling of Pol δ. With this in mind we have also analyzed the behavior of the Pol δ complexes on sparsely primed M13 DNA. The comparisons of the behavior of the Pol δ complexes reveal much greater differences. In this case, only the core+p68 trimer is able to perform synthesis of the 7.4 kb M13 in a manner comparable to the Pol δ4 holoenzyme. This suggests that the p68 subunit plays a major role in PCNA binding.

Our recent studies have established that the core+p68 trimer is a physiologically relevant enzyme, as the cellular content of Pol δ4 is wholly converted to the core+p68 trimer during challenge by genotoxic agents or by replication stress [30]. In other studies using the core+p68 trimer prepared by the methods reported here, we have shown that the core+p68 trimer exhibits altered properties which are consistent with the hypothesis that its formation may be useful to the cell undergoing genotoxic stress. The core+p68 trimer exhibits an increased discrimination against translesion synthesis across damaged templates, and a decreased tendency for extension of mismatched primers [44]. Pre-steady kinetic analyses further showed that the core+p68 enzyme has altered kinetic constants that are consistent with an increased proofreading ability, *viz*. greater fidelity in nucleotide incorporation than the Pol δ4 enzyme [45]. The question that arises is whether the core+p68 trimer is also present under other cellular conditions. Recent studies have reported that p12 expression at the mRNA and protein level is reduced in small lung cancer tissues and cell lines, and that siRNA depletion of p12 led to activation of cell cycle checkpoints, aberrant cell cycle progression and evidence for increased chromosomal breaks [46]. Depletion of p12 was also found to induce modest DNA damage, and an increased level of karyomere-like cells[47]. These findings indicate that p12 is required for normal DNA replication; however, it is not yet established whether the depletion of p12 results in the overall reduction of Pol δ4 holoenzyme levels, or whether this leads to a chronic elevation of the core+p68 trimer levels. This latter possibility could imply that the core+p68 enzyme could participate in chromosomal DNA replication, thereby causing DNA damage. Our findings that the core+p68 enzyme can sustain progressive DNA synthesis in the M13 assays supports a possibility that cells could survive by utilizing it for chromosomal DNA replication. In yeast, POL32, the yeast cognate of p68, is not essential although the deletion mutants exhibit slower growth and changes in sensitivity to DNA damage[18]. The questions of whether the Pol δ subassemblies may participate in DNA transactions, both in DNA replication and DNA repair, remains an open issue; our observations on the properties of the subassemblies do not eliminate all these possibilities. For example, our findings with the poly(dA)oligo(dT) substrate suggests that the core and the two trimers can sustain PCNA interaction and primer elongation at least in the kilobase range. This could be adequate for gap-filling synthesis in repair, and even in lagging strand synthesis which requires less than a kb of DNA synthesis.

As noted in the introduction, Pol δ, as isolated from calf thymus or human placenta, was originally characterized as a dimer of the p125 and p50 subunits [9]–[11]. This could be a consequence of the extensive series of chromatography steps required for their isolation that resulted in the removal of the p12 and p68 subunits, or possibly due to the loss of these subunits by proteolysis in spite of the use of protease inhibitors. To test the idea that the p12 and p68 subunits are more susceptible to proteolysis than the core enzyme, we examined the effects of human calpain-1 on Pol δ4. Both p68 and p12 were readily degraded by calpain-1, while the p125 and p50 subunits were resistant (Fig. 6). After the cleavage reaction, p68 was completely degraded with no detectable fragments, while p12 was almost completely degraded, with traces of two truncated products which were recognized by the polyclonal antibody against p12. These cleavages could be efficiently inhibited by the calpain inhibitor ALLN or calpeptin (Fig. 6). Thus, Pol δ could be converted *in vitro* from a heterotetramer to a core enzyme through the cleavage of p68 and p12 by calpain-1. The core enzyme appeared to be largely untouched, although it is possible that there might be nicking at the N or C-termini with losses that are not detected on SDS-PAGE. We have noted that the native and recombinant core+p68 trimers are highly unstable, unless they are rapidly purified and stored in liquid nitrogen. We cannot discount whether proteolytic activities in the preparations contribute to the loss of activity, although we note that the Pol δ4 holoenzymes isolated under similar conditions are not unstable (Fig. 4A).

The mammalian calpains consist of at least 15 calcium-dependent cysteine proteases which are members of a protein family that extends to lower eukaryotes and plants. Calpains are generally believed to regulate key cellular processes, that include differentiation, apoptosis, cell motility and cell cycle via signal-dependent cleavage of a limited set of substrate proteins [48], [49]. Like caspases, calpain substrates include a variety of cytoskeletal proteins such as the actin-binding protein fodrin as well as proteins involved in apoptosis such as Bax, p35, p53, pro-caspase 9, pro-caspase 3 and poly-ADP-ribose polymerase (PARP) [50]. Thus, the susceptibility of Pol δ subunits to cleavage by calpain-1 may have some potential cellular significance as it raises the possibility that Pol δ may be targeted as a substrate during apoptosis. Interestingly, both caspase-3 and calpain can mediate the cleavage of the human DNA pol ε catalytic subunit p261 to produce p140 *in vivo* and *in vitro* [51].

## References

[1] Garg P, Burgers PM (2005). DNA polymerases that propagate the eukaryotic DNA replication fork.. Crit Rev Biochem Mol Biol.

[2] Jeruzalmi D, O'Donnell M, Kuriyan J (2002). Clamp loaders and sliding clamps.. Curr Opin Struct Biol.

[3] Kesti T, Flick K, Keranen S, Syvaoja JE, Wittenberg C (1999). DNA polymerase epsilon catalytic domains are dispensable for DNA replication, DNA repair, and cell viability.. Mol Cell.

[4] Waga S, Stillman B (1998). The DNA replication fork in eukaryotic cells.. Annu Rev Biochem.

[5] Kunkel TA, Burgers PM (2008). Dividing the workload at a eukaryotic replication fork.. Trends Cell Biol.

[6] Nick McElhinny SA, Gordenin DA, Stith CM, Burgers PM, Kunkel TA (2008). Division of labor at the eukaryotic replication fork.. Mol Cell.

[7] Sancar A, Lindsey-Boltz LA, Unsal-Kacmaz K, Linn S (2004). Molecular mechanisms of mammalian DNA repair and the DNA damage checkpoints.. Annu Rev Biochem.

[8] Maloisel L, Fabre F, Gangloff S (2008). DNA polymerase delta is preferentially recruited during homologous recombination to promote heteroduplex DNA extension.. Mol Cell Biol.

[9] Lee MY, Tan CK, Downey KM, So AG (1984). Further studies on calf thymus DNA polymerase delta purified to homogeneity by a new procedure.. Biochemistry.

[10] Lee MY, Tan CK, Downey KM, So AG (1981). Structural and functional properties of calf thymus DNA polymerase delta.. Prog Nucleic Acid Res Mol Biol.

[11] Lee MY, Jiang YQ, Zhang SJ, Toomey NL (1991). Characterization of human DNA polymerase delta and its immunochemical relationships with DNA polymerase alpha and epsilon.. J Biol Chem.

[12] Lee MY, Tan CK, So AG, Downey KM (1980). Purification of deoxyribonucleic acid polymerase delta from calf thymus: partial characterization of physical properties.. Biochemistry.

[13] Mo J, Liu L, Leon A, Mazloum N, Lee MY (2000). Evidence that DNA polymerase delta isolated by immunoaffinity chromatography exhibits high-molecular weight characteristics and is associated with the KIAA0039 protein and RPA.. Biochemistry.

[14] Hughes P, Tratner I, Ducoux M, Piard K, Baldacci G (1999). Isolation and identification of the third subunit of mammalian DNA polymerase delta by PCNA-affinity chromatography of mouse FM3A cell extracts.. Nucleic Acids Res.

[15] Liu L, Mo J, Rodriguez-Belmonte EM, Lee MY (2000). Identification of a fourth subunit of mammalian DNA polymerase delta.. J Biol Chem.

[16] Li H, Xie B, Zhou Y, Rahmeh A, Trusa S (2006). Functional roles of p12, the fourth subunit of human DNA polymerase delta.. J Biol Chem.

[17] Xie B, Mazloum N, Liu L, Rahmeh A, Li H (2002). Reconstitution and characterization of the human DNA polymerase delta four-subunit holoenzyme.. Biochemistry.

[18] Gerik KJ, Li X, Pautz A, Burgers PM (1998). Characterization of the two small subunits of Saccharomyces cerevisiae DNA polymerase delta.. J Biol Chem.

[19] Zuo S, Bermudez V, Zhang G, Kelman Z, Hurwitz J (2000). Structure and activity associated with multiple forms of Schizosaccharomyces pombe DNA polymerase delta.. J Biol Chem.

[20] Prelich G, Tan CK, Kostura M, Mathews MB, So AG (1987). Functional identity of proliferating cell nuclear antigen and a DNA polymerase-delta auxiliary protein.. Nature.

[21] Zhang SJ, Zeng XR, Zhang P, Toomey NL, Chuang RY (1995). A conserved region in the amino terminus of DNA polymerase delta is involved in proliferating cell nuclear antigen binding.. J Biol Chem.

[22] Zhang P, Mo JY, Perez A, Leon A, Liu L (1999). Direct interaction of proliferating cell nuclear antigen with the p125 catalytic subunit of mammalian DNA polymerase delta.. J Biol Chem.

[23] Lu X, Tan CK, Zhou JQ, You M, Carastro LM (2002). Direct interaction of proliferating cell nuclear antigen with the small subunit of DNA polymerase delta.. J Biol Chem.

[24] Ducoux M, Urbach S, Baldacci G, Hubscher U, Koundrioukoff S (2001). Mediation of proliferating cell nuclear antigen (PCNA)-dependent DNA replication through a conserved p21(Cip1)-like PCNA-binding motif present in the third subunit of human DNA polymerase delta.. J Biol Chem.

[25] Wang Y, Zhang Q, Chen H, Li X, Mai W (2011). P50, the Small Subunit of DNA Polymerase Delta, Is Required for Mediation of the Interaction of Polymerase Delta Subassemblies with PCNA.. PLoS ONE.

[26] Xie B, Li H, Wang Q, Xie S, Rahmeh A (2005). Further characterization of human DNA polymerase delta interacting protein 38.. J Biol Chem.

[27] Podust VN, Chang LS, Ott R, Dianov GL, Fanning E (2002). Reconstitution of human DNA polymerase delta using recombinant baculoviruses: the p12 subunit potentiates DNA polymerizing activity of the four-subunit enzyme.. J Biol Chem.

[28] Harper JW, Elledge SJ (2007). The DNA damage response: ten years after.. Mol Cell.

[29] Branzei D, Foiani M (2010). Maintaining genome stability at the replication fork.. Nat Rev Mol Cell Biol.

[30] Zhang S, Zhou Y, Trusa S, Meng X, Lee EY (2007). A novel DNA damage response: rapid degradation of the p12 subunit of dna polymerase delta.. J Biol Chem.

[31] Berger I, Fitzgerald DJ, Richmond TJ (2004). Baculovirus expression system for heterologous multiprotein complexes.. Nat Biotechnol.

[32] Zhang P, Zhang SJ, Zhang Z, Woessner JFJ, Lee MY (1995). Expression and physicochemical characterization of human proliferating cell nuclear antigen.. Biochemistry.

[33] Jiang Y, Zhang SJ, Wu SM, Lee MY (1995). Immunoaffinity purification of DNA polymerase delta.. Arch Biochem Biophys.

[34] Trowitzsch S, Bieniossek C, Nie Y, Garzoni F, Berger I (2010). New baculovirus expression tools for recombinant protein complex production.. J Struct Biol.

[35] Vijayachandran LS, Viola C, Garzoni F, Trowitzsch S, Bieniossek C (2011). Robots, pipelines, polyproteins: enabling multiprotein expression in prokaryotic and eukaryotic cells.. J Struct Biol.

[36] Bieniossek C, Imasaki T, Takagi Y, Berger I (2012). MultiBac: expanding the research toolbox for multiprotein complexes.. Trends Biochem Sci.

[37] Xie S, Xie B, Lee MY, Dai W (2005). Regulation of cell cycle checkpoints by polo-like kinases.. Oncogene.

[38] Rahmeh AA, Zhou Y, Xie B, Li H, Lee EY (2012). Phosphorylation of the p68 Subunit of Pol delta Acts as a Molecular Switch To Regulate Its Interaction with PCNA.. Biochemistry.

[39] Acharya N, Klassen R, Johnson RE, Prakash L, Prakash S (2011). PCNA binding domains in all three subunits of yeast DNA polymerase delta modulate its function in DNA replication.. Proc Natl Acad Sci U S A.

[40] Zheng L, Shen B (2011). Okazaki fragment maturation: nucleases take centre stage.. J Mol Cell Biol.

[41] Henry RA, Balakrishnan L, Ying-Lin ST, Campbell JL, Bambara RA (2010). Components of the secondary pathway stimulate the primary pathway of eukaryotic Okazaki fragment processing.. J Biol Chem.

[42] Pascal JM, Tsodikov OV, Hura GL, Song W, Cotner EA (2006). A flexible interface between DNA ligase and PCNA supports conformational switching and efficient ligation of DNA.. Mol Cell.

[43] Dionne I, Nookala RK, Jackson SP, Doherty AJ, Bell SD (2003). A heterotrimeric PCNA in the hyperthermophilic archaeon Sulfolobus solfataricus.. Mol Cell.

[44] Meng X, Zhou Y, Zhang S, Lee EY, Frick DN (2009). DNA damage alters DNA polymerase delta to a form that exhibits increased discrimination against modified template bases and mismatched primers.. Nucleic Acids Res.

[45] Meng X, Zhou Y, Lee EY, Lee MY, Frick DN (2010). The p12 subunit of human polymerase delta modulates the rate and fidelity of DNA synthesis.. Biochemistry.

[46] Huang QM, Tomida S, Masuda Y, Arima C, Cao K (2010). Regulation of DNA polymerase POLD4 influences genomic instability in lung cancer.. Cancer Res.

[47] Huang QM, Akashi T, Masuda Y, Kamiya K, Takahashi T (2010). Roles of POLD4, smallest subunit of DNA polymerase delta, in nuclear structures and genomic stability of human cells.. Biochem Biophys Res Commun.

[48] Croall DE, DeMartino GN (1991). Calcium-activated neutral protease (calpain) system: structure, function, and regulation.. Physiol Rev.

[49] Croall DE, Ersfeld K (2007). The Calpains: modular design and functional diversity.. Genome Biol.

[50] Harwood SM, Yaqoob MM, Allen DA (2005). Caspase and calpain function in cell death: bridging the gap between apoptosis and necrosis.. Ann Clin Biochem.

[51] Liu W, Linn S (2000). Proteolysis of the human DNA polymerase epsilon catalytic subunit by caspase-3 and calpain specifically during apoptosis.. Nucleic Acids Res.

